# Sleep Deprivation and Its Impact on Cognitive Function and Academic Success in Health Sciences Students at Umm Al-Qura University

**DOI:** 10.7759/cureus.106708

**Published:** 2026-04-09

**Authors:** Rnad M Makkawi, Sadeem N Alnefaie, Aseel Aljuhani, Jana M Alshehri, Hams K Alzahrani, Jana Alsharif, Abeer Shaker

**Affiliations:** 1 College of Medicine, Umm Al-Qura University, Makkah, SAU; 2 Department of Pathology, College of Medicine, Umm Al-Qura University, Makkah, SAU

**Keywords:** academic performance, cognitive function, medical students, sleep deprivation, sleep quality

## Abstract

Background

Sleep deprivation is a growing concern among students, particularly in demanding fields such as health sciences, where it can impair cognitive function and academic performance. This study aimed to investigate the prevalence of sleep deprivation and its impact on health sciences students at Umm Al-Qura University.

Methods

A cross-sectional study was conducted during the 2024-2025 academic year, involving 211 undergraduate health sciences students. Data were collected through an online questionnaire assessing sociodemographic characteristics, sleep patterns, academic performance, stress levels, and electronic device use. Statistical analyses included independent t-tests, chi-square tests, and regression analyses to evaluate associations between sleep quality and academic outcomes.

Results

Among the participants, 102 (48.3%) reported difficulty sleeping. Poor sleep quality was significantly associated with shorter sleep duration, longer sleep latency, and irregular sleep schedules. Students with sleep difficulties were more likely to use digital devices before bedtime (72 (70.6%) vs. 56 (51.4%); p = 0.007) and to feel less refreshed upon waking (p = 0.001). Poor sleep quality scores were also significantly associated with poorer academic performance (p = 0.018), higher stress levels (p = 0.022), and difficulty completing academic tasks on time (p = 0.0039). Notably, 82 participants (38.86%) lacked awareness of recommended sleep duration.

Conclusion

This study highlights the high prevalence of sleep deprivation among health sciences students. Although sleep difficulties were commonly reported, the findings showed mixed associations with academic performance. Interventions focusing on reducing screen time, managing stress, and improving sleep education are recommended to enhance sleep quality. Addressing sleep deprivation is essential for improving student well-being and supporting academic success.

## Introduction

Sleep is an essential and active biological process that plays a vital role in various bodily functions, including growth, tissue repair, learning, and memory consolidation. Despite its importance, sleep deprivation has become increasingly common in modern society, as individuals often sacrifice sleep to meet daily demands. This behavior can lead to significant adverse outcomes, including daytime drowsiness and impairments in cognitive and psychomotor performance [1].

Ideally, adults should obtain 7-9 hours of sleep per night, while children and adolescents require even longer durations [2]. Chronic sleep deprivation, often referred to as “sleep debt,” can result in a range of negative effects, including reduced alertness, memory impairment, and decreased information processing capacity [2]. These consequences are particularly concerning for students, who frequently prioritize academic and social activities over adequate rest. Such patterns can impair cognitive functions essential for academic success, including attention, memory, and executive functions such as decision-making and problem-solving [3].

Evidence suggests that sleep deprivation disproportionately affects higher-order cognitive processes. While simpler tasks may remain relatively preserved, more complex functions, including creativity and emotional regulation, are significantly impaired. The prefrontal cortex is especially vulnerable to sleep loss, leading to diminished performance in tasks requiring planning, reasoning, and creative thinking [4]. Furthermore, sleep deprivation is associated with emotional disturbances, such as increased irritability and depressive symptoms, which can further compromise academic performance [5].

Medical students, in particular, face substantial academic workloads, competitive environments, and high expectations. Inadequate sleep in this group not only impairs cognitive function and academic performance but also contributes to physical fatigue, exhaustion, and reduced academic engagement. With the rapid advancement of technology and increased use of electronic devices, such as laptops, tablets, and smartphones, students are at heightened risk of sleep disturbances [6-8]. Numerous studies have demonstrated a negative association between sleep quality and academic performance, often reflected in lower grade point averages. This decline may be attributed not only to cognitive impairment but also to reduced motivation to engage in academic tasks due to fatigue [1].

Sleep medicine is an emerging field in Saudi Arabia, and there remains a scarcity of local university-based studies examining sleep problems among students, particularly those in health-related disciplines. Investigating sleep patterns in this population is important, as sleep deprivation may adversely affect future healthcare providers and the quality of healthcare delivery in the Kingdom of Saudi Arabia. Although several studies have explored sleep and health issues among university students in different regions, to our knowledge, limited research has specifically focused on sleep health among students in health science colleges [9,10]. Ensuring adequate sleep is therefore essential for maintaining cognitive performance, emotional well-being, and overall health, particularly in demanding academic and professional environments. As research continues to highlight the multifaceted impact of sleep deprivation, prioritizing sleep becomes increasingly important for optimal functioning in both personal and academic domains [9-11].

This study is guided by a conceptual framework suggesting that multiple sleep-related factors influence sleep quality among university students. These factors include sleep duration, sleep latency, irregular sleep schedules, and bedtime behaviors such as digital device use. Poor sleep quality may subsequently lead to increased stress, daytime fatigue, and reduced alertness, which can negatively affect academic performance and the ability to complete academic tasks efficiently.

Therefore, this study aims to investigate the prevalence of sleep deprivation and determine its impact on cognitive function among health sciences students at Umm Al-Qura University.

## Materials and methods

This cross-sectional study aimed to investigate the prevalence of sleep deprivation and its impact on cognitive function among health sciences students at Umm Al-Qura University, Makkah, Saudi Arabia. The target population included all undergraduate students enrolled in health-related colleges (Applied Medical Sciences, Medicine, Nursing, Pharmacy, and others) during the 2024-2025 academic year. A convenience sampling method was used to recruit participants, allowing the inclusion of students who were readily available and willing to participate during the study period. Eligible students were invited through classroom announcements, institutional emails, and social media platforms. Inclusion criteria comprised undergraduate health sciences students aged 18 years or older who provided informed consent and completed the questionnaire. Students who were not enrolled during the study period or who submitted incomplete questionnaires were excluded.

Data were collected using a self-administered online questionnaire developed by the authors. The final version was distributed via Google Forms (Google, Mountain View, CA, USA) in English to ensure accessibility and clarity. Although the questionnaire had not undergone formal psychometric validation or pilot testing, its content was reviewed by subject matter experts to ensure face validity and relevance to the study objectives. The questionnaire consisted of three sections. The first section collected sociodemographic information, including age, gender, year of study, and academic major. The second section assessed sleep patterns and habits, including sleep duration, sleep latency, and overall sleep quality. The third section focused on cognitive and academic performance, as well as related variables such as stress levels and the frequency and duration of electronic device use before bedtime.

Sleep deprivation was assessed based on participants’ self-reported sleep patterns and habits. Cognitive function and academic performance were also evaluated using self-reported measures. The full questionnaire is provided in Appendix 1.

Sample size determination

A total of 211 students completed the questionnaire and were included in the final analysis. The sample size was determined based on the number of respondents who met the inclusion criteria and provided complete data.

Ethical approval

This study was approved by the Biomedical Research Ethics Committee of the Faculty of Medicine, Umm Al-Qura University (Approval No. HAPO-02-K-012-2024-12-2394).

Informed consent

The purpose of the study was explained at the beginning of the questionnaire, and participation was entirely voluntary. All participants provided informed consent prior to data collection. Participant confidentiality and anonymity were maintained, and no identifying information was collected. Consent was obtained electronically from each participant.

Data analysis

Data analysis was performed using RStudio (Version 4.1.1; Posit, Boston, MA, USA). Descriptive statistics were used to summarize demographic characteristics, sleep variables, and academic performance. Continuous variables were presented as mean ± standard deviation (SD), while categorical variables were expressed as frequencies and percentages (N, %).

For comparisons between groups (students with and without sleep difficulties), independent t-tests and chi-square (χ²) tests were applied. Pearson’s correlation coefficient (r) was used to assess the relationships between sleep quality scores and variables such as academic performance, stress levels, and electronic device use. Multiple linear regression analysis was performed to identify predictors of sleep quality while controlling for relevant covariates. A p-value < 0.05 was considered statistically significant.

## Results

The current study included a total of 211 responses from eligible participants. As shown in Table 1, the majority of participants were aged 18-20 years (N = 175, 82.9%) and were in their first to third year of study (N = 198, 93.8%). Most participants were female (N = 184, 87.2%), and the most commonly reported field of study was medicine (N = 125, 59.2%). None of these baseline characteristics differed significantly between participants who reported sleep difficulties and those who did not.

**Table 1:** Comparing background data according to the reported difficulty sleeping. α = 0.05. p-values were obtained from Pearson's chi-square test of independence.

Term	Overall N (%)	With difficulty sleeping, N (%) (n = 102)	Without difficulty sleeping, N (%) (n = 109)	χ^2^	OR	p-value
Age group (years)						
18-20	175 (82.94)	82 (80.4)	93 (85.3)	4.2	0.6 (0.3-1.4)	0.239
21-23	33 (15.64)	19 (18.6)	14 (12.8)			
24-26	2 (0.95)	0 (0)	2 (1.8)			
27 and above	1 (0.47)	1 (1)	0 (0)			
Gender						
Male	27 (12.8)	17 (16.7)	10 (9.2)	2	2 (0.9-4.6)	0.155
Female	184 (87.2)	85 (83.3)	99 (90.8)			
Major						
Applied medical sciences	14 (6.64)	7 (6.9)	7 (6.4)	0.8	1.1 (0.4-3.3)	0.943
Medicine	125 (59.24)	60 (58.8)	65 (59.6)			
Nursing	10 (4.74)	6 (5.9)	4 (3.7)			
Other	40 (18.96)	18 (17.6)	22 (20.2)			
Pharmacy	22 (10.43)	11 (10.8)	11 (10.1)			
Study year						
1st year	22 (10.43)	7 (6.9)	15 (13.8)	6.7	0.5 (0.2-1.3)	0.153
2nd year	155 (73.46)	75 (73.5)	80 (73.4)			
3rd year	21 (9.95)	10 (9.8)	11 (10.1)			
4th year	5 (2.37)	4 (3.9)	1 (0.9)			
5th year or higher	8 (3.79)	6 (5.9)	2 (1.8)			

Among the various sleep-related complaints assessed, participants who reported difficulty sleeping were more likely to have difficulty waking up (N = 53 (52.0%) vs. N = 73 (67.0%); p = 0.037), to use digital devices before bedtime (N = 72 (70.6%) vs. N = 56 (51.4%); p = 0.007), and to have irregular sleep schedules (N = 64 (62.7%) vs. N = 47 (43.1%); p = 0.007) (Appendix 2).

As shown in Table 2, participants with sleep difficulties were more likely to have shorter weekday sleep duration (p = 0.012) and were less likely to feel refreshed upon waking (p = 0.001). Additionally, 20 participants (19.6%) in this group reported prolonged sleep latency of more than 60 minutes, whereas none of the participants without sleep difficulties reported this (p < 0.001). Participants with sleep difficulties also reported a significantly higher frequency of sleep problems, particularly occurring on four or more nights per week (p = 0.001). Although daytime fatigue was more commonly reported among those with sleep difficulties (86, 84.3%) compared to those without (79, 72.5%), this difference did not reach statistical significance (p = 0.056). Overall, 135 participants (63.98%) reported going to bed late on 1-3 nights per week. Wake-up time did not differ significantly between participants with and without sleep difficulties (p = 0.247).

**Table 2:** Comparing sleeping habits according to the reported difficulty sleeping. α = 0.05. *p < 0.05, **p < 0.01, ***p < 0.001. p-values were obtained from Pearson's chi-square test of independence.

Term	Overall N (%)	With difficulty sleeping, N (%) (n = 102)	Without difficulty sleeping, N (%) (n = 109)	χ^2^	OR	p-value
Sleep hours on weekdays						
Less than 4 hours	15 (7.11)	13 (12.7)	2 (1.8)	12.9	5.9 (1.2-28.3)	0.012*
4-5 hours	61 (28.91)	32 (31.4)	29 (26.6)			
6-7 hours	75 (35.55)	35 (34.3)	40 (36.7)			
8-9 hours	41 (19.43)	14 (13.7)	27 (24.8)			
More than 9 hours	19 (9)	8 (7.8)	11 (10.1)			
Refreshed and rested upon waking						
Never	12 (5.69)	8 (7.8)	4 (3.7)	19.9	0.7 (0.2-2.7)	0.001**
Rarely	44 (20.85)	33 (32.4)	11 (10.1)			
Sometimes	100 (47.39)	40 (39.2)	60 (55)			
Most of the time	44 (20.85)	18 (17.6)	26 (23.9)			
Always	11 (5.21)	3 (2.9)	8 (7.3)			
Sleep latency						
Less than 10 minutes	24 (11.37)	1 (1)	23 (21.1)	47.4	0.1 (0-0.5)	<0.001***
10-30 minutes	104 (49.29)	42 (41.2)	62 (56.9)			
30-60 minutes	63 (29.86)	39 (38.2)	24 (22)			
More than 60 minutes	20 (9.48)	20 (19.6)	0 (0)			
Late for bedtime						
Once a week	23 (10.9)	8 (7.8)	15 (13.8)	1.9	0.5 (0.2-1.4)	0.387
2-3 times a week	112 (53.08)	56 (54.9)	56 (51.4)			
4 or more times a week	76 (36.02)	38 (37.3)	38 (34.9)			
Wake-up time on weekdays						
Pre-dawn (before 5:00 AM)	12 (5.69)	6 (5.9)	6 (5.5)	6.7	1.4 (0.4-4.6)	0.247
Early morning (5:00-7:00 AM)	95 (45.02)	40 (39.2)	55 (50.5)			
Mid-morning (7:00-9:00 AM)	38 (18.01)	25 (24.5)	13 (11.9)			
Late morning (9:00-11:00 AM)	32 (15.17)	16 (15.7)	16 (14.7)			
In the afternoon (after 12:00 PM)	12 (5.69)	6 (5.9)	6 (5.5)			
I don't have a fixed time to wake up	22 (10.43)	9 (8.8)	13 (11.9)			
Tired or fatigued during the day						
Yes	165 (78.2)	86 (84.3)	79 (72.5)	3.7	2 (1-4)	0.056
No	46 (21.8)	16 (15.7)	30 (27.5)			
Frequency of sleep problems						
I haven’t experienced any problems	22 (10.43)	7 (6.9)	15 (13.8)	22	2.1 (0.6-8)	0.001**
One night	28 (13.27)	5 (4.9)	23 (21.1)			
Two nights	51 (24.17)	22 (21.6)	29 (26.6)			
Three nights	48 (22.75)	28 (27.5)	20 (18.3)			
Four nights	28 (13.27)	19 (18.6)	9 (8.3)			
5-7 nights	34 (16.11)	21 (20.6)	13 (11.9)			

Table 3 shows that participants who reported sleeping difficulties were comparable to those who did not across several sleep-related factors, including the use of sleep aids (p = 0.69), coping strategies for sleep deprivation (p = 0.18), types of caffeinated beverages consumed (p = 0.39), and frequency of consumption (p = 0.93). Interestingly, the prevalence of a diagnosed sleep disorder did not differ significantly between the two groups (p = 0.80), which may suggest potential underdiagnosis of sleep disorders. In contrast, participants with reported sleeping difficulties were more likely to report poorer sleep quality (p = 0.018), longer duration of sleep problems (p < 0.001), and insufficient sleep (N = 72 (70.6%) vs. N = 55 (50.5%); p = 0.004).

**Table 3:** Comparing reported sleeping details according to the reported difficulty sleeping. α = 0.05. *p < 0.05, **p < 0.01, ***p < 0.001. p-values were obtained from Pearson's chi-square test of independence.

Term	Overall N (%)	With difficulty sleeping, N (%) (n = 102)	Without difficulty sleeping, N (%) (n = 109)	χ^2^	OR	p-value
Use sleep aids						
Yes	28 (13.27)	15 (14.7)	13 (11.9)	0.2	1.3 (0.6-2.8)	0.695
No	183 (86.73)	87 (85.3)	96 (88.1)			
Coping strategy for sleep deprivation						
Caffeine consumption	52 (24.64)	31 (30.4)	21 (19.3)	7.5	2.6 (0.9-7.2)	0.183
Exercise	22 (10.43)	8 (7.8)	14 (12.8)			
Meditation or relaxation techniques	21 (9.95)	11 (10.8)	10 (9.2)			
Napping	43 (20.38)	16 (15.7)	27 (24.8)			
None	66 (31.28)	34 (33.3)	32 (29.4)			
Other	7 (3.32)	2 (2)	5 (4.6)			
Caffeinated beverages consumed						
Coffee	117 (55.45)	63 (61.8)	54 (49.5)	5.2	1.2 (0.2-8.6)	0.39
Energy drinks	4 (1.9)	2 (2)	2 (1.8)			
Matcha	18 (8.53)	8 (7.8)	10 (9.2)			
Other	27 (12.8)	10 (9.8)	17 (15.6)			
Soft drinks	10 (4.74)	6 (5.9)	4 (3.7)			
Tea	35 (16.59)	13 (12.7)	22 (20.2)			
Number of cups of caffeinated beverages consumed per day						
1	125 (59.24)	59 (57.8)	66 (60.6)	0.4	1 (0.5-1.8)	0.937
2	54 (25.59)	26 (25.5)	28 (25.7)			
3	22 (10.43)	12 (11.8)	10 (9.2)			
4 and more	10 (4.74)	5 (4.9)	5 (4.6)			
Diagnosed with a sleep disorder						
Yes	25 (11.85)	11 (10.8)	14 (12.8)	0.1	0.8 (0.4-1.9)	0.803
No	186 (88.15)	91 (89.2)	95 (87.2)			
Relationships, mood, or energy affected by poor sleep						
Never	23 (10.9)	11 (10.8)	12 (11)	4.8	1.4 (0.5-3.8)	0.304
Slightly	48 (22.75)	19 (18.6)	29 (26.6)			
Sometimes	75 (35.55)	40 (39.2)	35 (32.1)			
Much	33 (15.64)	13 (12.7)	20 (18.3)			
Very much	32 (15.17)	19 (18.6)	13 (11.9)			
Productivity and focus affected by poor sleep						
Never	12 (5.69)	7 (6.9)	5 (4.6)	1.8	1.3 (0.3-4.7)	0.764
Slightly	38 (18.01)	20 (19.6)	18 (16.5)			
Sometimes	72 (34.12)	33 (32.4)	39 (35.8)			
Much	47 (22.27)	20 (19.6)	27 (24.8)			
Very much	42 (19.91)	22 (21.6)	20 (18.3)			
Duration of sleep problems						
I have no problems sleeping	59 (27.96)	17 (16.7)	42 (38.5)	21.7	0.5 (0.2-1.1)	<0.001***
<3 months	68 (32.23)	30 (29.4)	38 (34.9)			
3-6 months	23 (10.9)	12 (11.8)	11 (10.1)			
7-12 months	15 (7.11)	10 (9.8)	5 (4.6)			
More than a year	46 (21.8)	33 (32.4)	13 (11.9)			
Family history of sleep disorders						
Yes	33 (15.64)	13 (12.7)	20 (18.3)	0.9	0.7 (0.3 to 1.4)	0.352
No	178 (84.36)	89 (87.3)	89 (81.7)			
Perceived quality of sleep						
Very bad	8 (3.79)	7 (6.9)	1 (0.9)	11.9	4.5 (0.5-39.4)	0.018*
Bad	56 (26.54)	34 (33.3)	22 (20.2)			
Normal	95 (45.02)	42 (41.2)	53 (48.6)			
Good	38 (18.01)	14 (13.7)	24 (22)			
Very good	14 (6.64)	5 (4.9)	9 (8.3)			
Getting enough sleep						
Yes	84 (39.81)	30 (29.4)	54 (49.5)	8.1	0.4 (0.2-0.7)	0.004**
No	127 (60.19)	72 (70.6)	55 (50.5)			

Table 4 describes the academic performance of the study participants. Most participants rated their performance as average (N = 81, 38.39%) or good (N = 77, 36.49%). The majority also reported that lack of sleep affected their academic performance (N = 128, 60.66%). However, none of the academic performance indicators, including self-reported grades, study focus, or difficulty understanding new information, were significantly associated with the presence or absence of sleep difficulties (p > 0.05).

**Table 4:** Comparing basic academic performance data according to the reported difficulty sleeping. α = 0.05. *p < 0.05, **p < 0.01, ***p < 0.001. p-values were obtained from Pearson's chi-square test of independence.

Term	Overall N (%)	With difficulty sleeping, N (%) (n = 102)	Without difficulty sleeping, N (%) (n = 109)	χ^2^	OR	p-value
Overall academic performance						
Poor	5 (2.37)	3 (2.9)	2 (1.8)	2.8	2.1 (0.3-13.1)	0.42
Average	81 (38.39)	34 (33.3)	47 (43.1)			
Good	77 (36.49)	38 (37.3)	39 (35.8)			
Excellent	48 (22.75)	27 (26.5)	21 (19.3)			
Lack of sleep affects academic performance						
Yes	128 (60.66)	58 (56.9)	70 (64.2)	1.3	0.8 (0.4-1.4)	0.534
Maybe	65 (30.81)	34 (33.3)	31 (28.4)			
No	18 (8.53)	10 (9.8)	8 (7.3)			
Good grades on exams						
Rarely	11 (5.21)	5 (4.9)	6 (5.5)	0.3	1 (0.3-3.5)	0.841
Sometimes	74 (35.07)	34 (33.3)	40 (36.7)			
Always	126 (59.72)	63 (61.8)	63 (57.8)			
Stressed due to studies						
Rarely	4 (1.9)	1 (1)	3 (2.8)	3.4	0.4 (0-4.5)	0.338
Sometimes	51 (24.17)	22 (21.6)	29 (26.6)			
Often	67 (31.75)	30 (29.4)	37 (33.9)			
Always	89 (42.18)	49 (48)	40 (36.7)			
Specific academic goals						
Rarely	24 (11.37)	12 (11.8)	12 (11)	0	1.1 (0.4-3)	0.998
Sometimes	44 (20.85)	21 (20.6)	23 (21.1)			
Often	52 (24.64)	25 (24.5)	27 (24.8)			
Always	91 (43.13)	44 (43.1)	47 (43.1)			
Focus while studying						
Poor	22 (10.43)	11 (10.8)	11 (10.1)	2.8	1 (0.4-2.5)	0.422
Average	106 (50.24)	53 (52)	53 (48.6)			
Good	71 (33.65)	30 (29.4)	41 (37.6)			
Excellent	12 (5.69)	8 (7.8)	4 (3.7)			
Difficulty understanding new information						
Rarely	25 (11.85)	11 (10.8)	14 (12.8)	4.4	0.9 (0.4-2.3)	0.217
Sometimes	117 (55.45)	53 (52)	64 (58.7)			
Often	53 (25.12)	32 (31.4)	21 (19.3)			
Always	16 (7.58)	6 (5.9)	10 (9.2)			
Problem-solving skills						
Poor	16 (7.58)	10 (9.8)	6 (5.5)	1.9	1.7 (0.6-5)	0.587
Average	84 (39.81)	42 (41.2)	42 (38.5)			
Good	90 (42.65)	40 (39.2)	50 (45.9)			
Excellent	21 (9.95)	10 (9.8)	11 (10.1)			
Difficulty with important academic decisions						
Rarely	48 (22.75)	25 (24.5)	23 (21.1)	1.4	1.3 (0.6-2.5)	0.708
Sometimes	95 (45.02)	44 (43.1)	51 (46.8)			
Often	51 (24.17)	23 (22.5)	28 (25.7)			
Always	17 (8.06)	10 (9.8)	7 (6.4)			

Similarly, Table 5 shows that the perceived relationship between sleep and academic performance was comparable between participants who reported sleep difficulties and those who did not (p > 0.05). However, notable findings include that only 129 participants (61.14%) were aware of the recommended sleep duration. This is particularly concerning, given that the majority of participants were medical students. Lack of sleep was reported to affect the timely completion of academic tasks, with 48.8% of participants indicating that it often affected them, 33.65% reporting that it always affected them, and 17.5% reporting that it rarely affected them. Although these findings were similar between both groups, they highlight the need to increase awareness and promote healthy sleep habits within the target population. The sleep quality score was based on 10 items and ranged from -10 to 10. Among participants who reported sleep difficulties, the mean score was -0.55 (range: -10 to 8), whereas those without sleep difficulties had a higher mean score of 3.46 (Appendix 3).

**Table 5:** Comparing perceived relationship between sleep and academic performance according to the reported difficulty sleeping. α = 0.05. *p < 0.05, **p < 0.01, ***p < 0.001. p-values were obtained from Pearson's chi-square test of independence.

Term	Overall N (%)	With difficulty sleeping, N (%) (n = 102)	Without difficulty sleeping, N (%) (n = 109)	χ^2^	OR	p-value
Know the recommended hours of sleep						
Yes	129 (61.14)	67 (65.7)	62 (56.9)	1.4	1.5 (0.8-2.5)	0.242
No	82 (38.86)	35 (34.3)	47 (43.1)			
Lack of sleep and difficulty completing academic tasks on time						
Rarely	37 (17.54)	15 (14.7)	22 (20.2)	1.7	0.8 (0.4-1.6)	0.43
Often	103 (48.82)	49 (48)	54 (49.5)			
Always	71 (33.65)	38 (37.3)	33 (30.3)			
Poor sleep fatigue affects academic performance outside the classroom						
Affects significantly	59 (27.96)	28 (27.5)	31 (28.4)	0.7	0.9 (0.5-1.7)	0.876
Affects somewhat	115 (54.5)	58 (56.9)	57 (52.3)			
Does not affect much	33 (15.64)	14 (13.7)	19 (17.4)			
Does not affect at all	4 (1.9)	2 (2)	2 (1.8)			
Poor sleep and academic motivation						
Significantly reduces motivation	71 (33.65)	32 (31.4)	39 (35.8)	5.3	0.7 (0.4-1.4)	0.15
Somewhat reduces motivation	105 (49.76)	55 (53.9)	50 (45.9)			
Does not affect motivation much	24 (11.37)	13 (12.7)	11 (10.1)			
Does not affect motivation at all	11 (5.21)	2 (2)	9 (8.3)			
Focus after a good night's sleep						
My focus is much better with good sleep	143 (67.77)	65 (63.7)	78 (71.6)	4.1	0.8 (0.4-1.4)	0.249
My focus is somewhat better with good sleep	54 (25.59)	28 (27.5)	26 (23.9)			
No difference in focus	11 (5.21)	6 (5.9)	5 (4.6)			
I focus better with less sleep	3 (1.42)	3 (2.9)	0 (0)			

Table 6 shows the association between various covariates and sleep quality scores. Poor academic performance was significantly associated with poorer sleep quality (mean score: -3.8; p = 0.018). Experiencing stress due to academic studies was also significantly associated with sleep quality (p = 0.022). Additionally, difficulty completing academic tasks on time due to poor sleep was significantly associated with poorer sleep quality (p = 0.0039).

**Table 6:** Association between perceived sleep quality and reported academic performance.

Perceived sleep quality versus:	p-value	Test statistic	Method
Gender	0.1868	1.347	Welch two-sample t-test
Major	0.8674	0.316	One-way ANOVA
Academic performance overall	0.0181*	3.425	
Lack of sleep affects academic performance	0.7762	0.254	One-way ANOVA
Good grades on exams	0.2392	1.44	
Stressed due to your studies	0.0220*	3.277	
Specific academic goals	0.6	0.624	One-way ANOVA
Focus while studying	0.0789	2.295	
Difficulty understanding new information	0.1575	1.752	
Problem-solving skills	0.927	0.154	
Difficulty with important academic decisions	0.1729	1.678	
Know recommended hours of sleep	0.6342	0.477	Welch two-sample t-test
Lack of sleep and difficulty completing academic tasks on time	0.0039**	5.707	One-way ANOVA
Poor sleep fatigue affects academic performance outside the classroom	0.1868	1.616	One-way ANOVA
Poor sleep and academic motivation	0.8032	0.331	
Focus after a good night's sleep	0.4027	0.981	

While no significant association was found between academic performance and the presence of self-reported sleeping difficulties (Table 4), a significant association was observed between academic performance and sleep quality scores (Table 6). 

## Discussion

Sleep plays a crucial role in learning and memory consolidation. It is essential for the formation of synapses between dendritic branches, which facilitate the retention of learned information, enabling students to recall it more efficiently and over longer periods [12,13]. This cross-sectional study assessed the prevalence and awareness of sleep deprivation among health sciences students and its impact on concentration, memory, and academic performance. A secondary objective was to identify the causes of sleep deprivation, compare well-rested and sleep-deprived students, and explore challenges arising from insufficient sleep. A total of 211 participants completed the online questionnaire, of whom 102 (48.3%) reported difficulty sleeping. Most participants were medical students (N = 125, 59.24%) and were in their second year of study (N = 155, 73.46%). Students with difficulty sleeping were more likely to sleep fewer than four hours on weekdays (N = 13 (12.7%) vs. N = 2 (1.8%)) and demonstrated significantly longer sleep latency and higher odds of reporting irregular sleep schedules. Approximately 77 participants (35.55%) of the total sample reported sleeping 6-7 hours on weekdays, which is below the recommended threshold of seven or more hours as defined by the American Academy of Sleep Medicine and the Sleep Research Society [14]. Previous studies have reported similar findings, supporting the high prevalence of sleep deprivation among healthcare students [15-17]. Zeek et al. reported that more than 50% of pharmacy students obtained less than seven hours of sleep during weekdays [17]. Another study among medical officers in Malaysia found that 166 (78.64%) participants experienced sleep deprivation, which negatively affected their work performance compared to those with adequate sleep patterns [18].

Digital device use was more frequently reported among participants with sleep difficulties (N = 72 (70.6%) vs. N = 56 (51.4%)). This finding is consistent with previous studies demonstrating a negative association between digital device use and sleep quality [19-22]. No significant differences were observed regarding caffeine consumption, exposure to noisy environments, or the presence of medical conditions. Overall, 38.86% (N = 82) of participants reported a lack of awareness of the recommended sleep duration. The majority of participants (N = 115, 54.5%) reported that fatigue due to poor sleep somewhat affected their academic performance. Similarly, 83.41% (N = 176) indicated that poor sleep reduced their academic motivation. In contrast, 93.36% (N = 197) reported improved concentration and focus after a good night’s sleep.

Among participants with sleep difficulties, the mean perceived sleep quality score was -0.55 (SD = 3.47). In contrast, participants without sleep difficulties had a higher mean score of 3.46 (SD = 3.26). Significant findings from the analysis showed that poor academic performance was associated with worse perceived sleep quality scores, whereas excellent academic performance was associated with better sleep quality. Students experiencing higher levels of academic stress also reported poorer sleep quality. Additionally, lack of sleep was associated with greater difficulty in completing academic tasks on time, particularly among those who rarely obtained sufficient sleep. However, no significant associations were observed between sleep quality and other factors such as gender, academic major, or awareness of recommended sleep duration. Overall, while sleep quality was associated with perceived academic outcomes, other factors such as focus, motivation, and fatigue showed less direct associations. Previous studies have similarly examined the relationship between sleep patterns and academic performance, reporting consistent findings. Zeek et al. reported that longer sleep duration the night prior to examinations was associated with higher GPAs [17]. Medeiros et al. also found that medical students with longer sleep duration achieved higher examination scores [23]. Similarly, Veldi et al. reported a significant association between sleep behaviors and academic progression [24]. These findings are further supported by several cross-sectional studies [15,23-27]. In the present study, 187 (88.636%) participants reported sleep latency of more than 10 minutes. Agu et al. observed that students with longer sleep latency had poorer academic performance compared to those with shorter sleep latency [28]. Furthermore, the present findings are consistent with previous reports linking stress and sleep patterns. Almojali et al. found that medical students without stress were less likely to experience poor sleep quality [29]. The relationship between sleep patterns and stress is bidirectional, forming a reinforcing cycle [29,30]. Ayalew et al. also reported that coffee consumption, stress, and anxiety were significantly associated with sleep deprivation [16].

This study assessed the awareness and prevalence of sleep deprivation among health sciences students and its impact on concentration, memory, and academic performance. The findings highlight a high prevalence of sleep-related issues among healthcare students. In addition, participants’ awareness of the importance of regular and sufficient sleep was suboptimal. The study also demonstrated an association between digital device use and sleep problems. Furthermore, a significant relationship was observed between sleep quality and academic performance, as students with inadequate sleep reported higher stress levels and greater difficulty in completing academic tasks on time. These findings underscore the need to raise awareness of the importance of high-quality sleep as a key factor in improving academic outcomes among healthcare students. Accordingly, attention is warranted from both students and academic administrators. Promoting healthy sleep behaviors, stress management strategies, and reduced digital device use may help establish better sleep habits, ultimately improving productivity and quality of life. Further research is needed to explore additional causes of sleep deprivation beyond digital device use, such as academic pressure and lifestyle-related factors, to provide a more comprehensive understanding of the issue. Investigating the role of sleep hygiene education and its impact on sleep quality among health sciences students may also be beneficial.

Limitations

Several limitations of this study should be acknowledged. The cross-sectional design does not allow for the establishment of causal relationships between sleep deprivation and its effects on academic performance. In addition, self-reported measures of sleep quality and academic performance may be subject to bias due to participants’ subjective perceptions and may differ from findings obtained using objective assessments. Furthermore, the study did not fully account for potential confounding variables, such as pre-existing mental health conditions or diagnosed sleep disorders, which may have influenced the observed associations between sleep deprivation and the other studied variables.

## Conclusions

This study demonstrated a high prevalence of sleep deprivation among health sciences students. Although sleep difficulties were commonly reported, the findings showed mixed associations with academic performance. These results underscore the importance of sleep quality for cognitive function, memory, and concentration, and highlight the potential benefits of sleep education programs. Frequent use of digital devices before bedtime may contribute to poorer sleep quality, indicating the need for strategies aimed at reducing screen time prior to sleep.

## Appendices

Appendix 1

Questionnaire

Section 1: Demographic information

1. What is your age?

18-20

21-23

24-26

27 and above

2. What is your gender?

Female 

Male 

3. What is your major?

Medicine

Dentistry

Nursing

Midwifery

Public health

Health information management

Pharmacy

Physiotherapy

Respiratory therapy

Nutrition

Applied medical sciences

Public health and health information management

Other

4. What year are you currently in?

First year

Second year

Third year

Fourth year

Fifth year or higher

Section 2: Sleep patterns and habits

5. How many hours of sleep do you usually get on weekdays?

Less than 4 hours

4-5 hours

6-7 hours

8-9 hours

More than 9 hours

6. Do you feel refreshed and rested when you wake up in the morning?

Always

Most of the time

Sometimes

Rarely

Never

7. What time do you usually go to bed on weekdays?

Between 7 and 9 PM

Between 10 and 12 PM

Between 1 and 3 AM

8. How long does it typically take you to fall asleep?

Less than 10 minutes

10-30 minutes

30-60 minutes

More than 60 minutes

9. How many times a week are you late for bedtime?

Once a week

2-3 times a week

4 or more times a week

10. What problems do you encounter while sleeping? (Please select all that apply)

Difficulty sleeping

Waking up in the middle of the night

Intermittent sleep

Difficulty waking up

11. How many nights per week do you experience problems with your sleep?

One night

Two nights

Three nights

Four nights

5-7 nights

I haven’t experienced any problems

12. What time do you usually wake up on weekdays?

Pre-dawn (before 5:00 AM)

Early morning (5:00-7:00 AM)

Mid-morning (7:00-9:00 AM)

Late morning (9:00-11:00 AM)

In the afternoon (after 12:00 PM)

I don't have a fixed time to wake up

13. Do you often feel tired or fatigued during the day?

Yes

No

14. Which of the following factors affect your sleep patterns? (Please select all that apply)

Stress

Caffeine consumption

Digital devices (such as phones and computers)

Irregular sleep schedule

Noise

Environment (such as temperature and lighting)

Medical conditions

Other

15. Do you use any sleep aids (such as medications or herbal remedies)? 

Yes

No

16. What strategies do you use to cope with sleep deprivation? 

Caffeine consumption

Napping

Exercise

Meditation or relaxation techniques

None

Other

17. What types of caffeinated beverages do you usually drink? 

Coffee

Tea

Energy drinks

Soft drinks

Matcha

Other

18. How many cups of coffee or caffeinated beverages do you consume daily? 

1

2

3

4 and more 

19. Have you ever been diagnosed with a sleep disorder (e.g., insomnia, sleep apnea)? 

Yes

No

20. How much have your relationships, mood, and energy been affected by your poor sleep?

Never

Slightly

Sometimes

Much

Very much

21. How much have your productivity, focus, and ability to stay awake been affected by your poor sleep? 

Never

Slightly

Sometimes

Much

Very much

22. How long have you had problems sleeping? 

I have no problems sleeping

I had problems in less than a month.

1-2 months

3-6 months

7-12 months

More than a year

23. Is there a family history of sleep disorders? 

Yes 

No 

24. How do you rate the quality of your sleep?

Very good

Good

Normal

Bad

Very bad

Section 3: Cognitive and academic performance 

25. Do you think you are getting enough sleep?

Yes

No

26. How would you describe your academic performance overall?

Poor

Average

Good

Excellent

27. Do you believe that lack of sleep affects your academic performance? 

Yes

Maybe

No

28. Do you often achieve good grades on your exams? 

Yes

Sometimes

Rarely

No

29. How often do you feel stressed due to your studies?

Always

Often

Sometimes

Rarely

30. Do you set specific academic goals for yourself?

Always

Often

Sometimes

Rarely

31. How would you assess your ability to focus while studying? 

Poor

Average

Good

Excellent

32. How often do you feel that you have difficulty understanding new information?

Always

Often

Sometimes

Rarely

33. How would you describe your problem-solving skills? 

Weak

Average

Good

Excellent

34. Do you find it difficult to make important academic decisions? 

Always

Often

Sometime

Rarely

35. Are you aware of the recommended hours of sleep for college students? 

Yes

No

36. Do you feel that lack of sleep makes it difficult for you to complete your academic tasks on time? 

Always

Often

Rarely

Never

37. To what extent do you think the fatigue caused by lack of sleep affects your performance in academic activities outside the classroom? 

Affects significantly

Affects somewhat

Does not affect much

Does not affect at all

38. How does lack of sleep affect your motivation to complete assignments and prepare for exams? 

Significantly reduces motivation

Somewhat reduces motivation

Does not affect motivation much

Does not affect motivation at all

39. How do you evaluate your ability to focus and engage with new information when you’ve had a good night's sleep compared to days when you suffer from lack of sleep?

My focus is much better with good sleep

My focus is somewhat better with good sleep

No difference in focus

I focus better with less sleep

Appendix 2

**Table 7:** Comparing certain sleep-time problems according to the reported difficulty sleeping. α = 0.05. *p < 0.05, **p < 0.01, ***p < 0.001. p-values were obtained from Pearson's chi-square test of independence.

Term	Overall N (%)	Difficulty sleeping		
		Yes, N (%) (n = 102)	No, N (%) (n = 109)	p-value
Difficulty waking up				0.037*
Yes	126 (59.72)	53 (52)	73 (67)	
No	85 (40.28)	49 (48)	36 (33)	
Intermittent sleep				0.221
Yes	77 (36.49)	42 (41.2)	35 (32.1)	
No	134 (63.51)	60 (58.8)	74 (67.9)	
Caffeine consumption				0.346
Yes	79 (37.44)	42 (41.2)	37 (33.9)	
No	132 (62.56)	60 (58.8)	72 (66.1)	
Digital devices (such as phones and computers)				0.007**
Yes	128 (60.66)	72 (70.6)	56 (51.4)	
No	83 (39.34)	30 (29.4)	53 (48.6)	
Environment (such as temperature and lighting)				0.444
Yes	79 (37.44)	35 (34.3)	44 (40.4)	
No	132 (62.56)	67 (65.7)	65 (59.6)	
Other				0.82
Yes	27 (12.8)	12 (11.8)	15 (13.8)	
No	184 (87.2)	90 (88.2)	94 (86.2)	
Irregular sleep schedule				0.007**
Yes	111 (52.61)	64 (62.7)	47 (43.1)	
No	100 (47.39)	38 (37.3)	62 (56.9)	
Noise				0.99
Yes	72 (34.12)	35 (34.3)	37 (33.9)	
No	139 (65.88)	67 (65.7)	72 (66.1)	
Medical conditions				0.146
Yes	25 (11.85)	16 (15.7)	9 (8.3)	
No	186 (88.15)	86 (84.3)	100 (91.7)	
Stress				0.178
Yes	171 (81.04)	87 (85.3)	84 (77.1)	
No	40 (18.96)	15 (14.7)	25 (22.9)	

Appendix 3 

**Table 8:** Sleep quality scores according to the reported difficulty sleeping.

Overall sample			
Term	Avg (SD)	Median (IQR)	Range: min-max
Perceived sleep quality	1.52 (3.91)	2 (6)	-10 to 10
Difficulty sleeping: yes	-0.55 (3.47)	-1 (4)	-10 to 8
Difficulty sleeping: no	3.46 (3.26)	4 (4)	-1 to 10
